# Smoking Tobacco

**Published:** 1880-09

**Authors:** 


**SMOKING TOBACCO.**—There is no vice of the appetite which does not find advocates among otherwise respectable people. The *Boston Medical and Surgical Journal*, the standard New-England medical periodical, is quoted as advocating smoking tobacco as a preventive of clergyman's sore throat. Now, some twenty years of personal observation with multitudes of confessions from the suffering, prove that smoking cigars is the immediate excitant of some of the most fearful sufferings ever recorded in medical books connected with the throat, an actual slow starving to death, from the utter inability to swallow a particle of nutriment, solid or fluid!

Again. Three fourths of all cases of throat trouble, put down under the head of Chronic Laryngitis, or Clergymen's Sore Throat, as eminent medical men of all schools admit, arise from a wrong condition of the digestive functions; in plainer language, clergymen's sore throat is generally the result of injudicious eating, of a disproportion between exercise and diet That a man may feel better, or rather is less sensible of discomfort in his throat after smoking a cigar or pipe, is admitted so would he from a dose of opium; but the effect of both is to obtund the sensibility of the parts, to place the patient falsely at ease, while the malady is burrowing in the system, gathering greater power, requiring more and more tobacco to keep the patient comfortable, until it refuses to be kept under control any longer, and either surprises the patient by death from starvation, or forcing some other outlet, assumes a new form of disease, and the patient "was cured of his laryngitis by smoking cigars," but died afterwards of something else! The truth is, neither opium, nor "liquor," nor tobacco, ever of themselves cured any body of any thing since the world began.

Dupuytren, one of the most eminent and honored names in medicine, says of tobacco-smoking: "I can not understand the progress of this filthy custom among educated people. It is incredible that a man of liberal education should consent, thus deliberately to debase his intellect; that a man who has enjoyed the pleasures of literary and scientific information should prefer to the sublime enjoyments of the mind the ignoble pleasure of rendering himself disgusting to all around him."

The *London Lancet*, high authority in the medical world, says, that "dividing the young gentlemen who attend the Polytechnic School at Paris into smokers and non-smokers, not only do the smokers on entering the classes take a lower rank, but in all the examinations afterwards the average rank of the smokers constantly falls, while those who do not smoke at all, enjoy a clearness of brain and thought of which the smokers have no experience." We can not accept this as demonstrative of the fact that smoking tobacco does of itself certainly have a marked deleterious impression on the mental faculties, although it strongly points that way. If, however, that solution is rejected, it follows that young fellows who smoke cigars, either lack the sense or the application necessary to reach positions of enviable distinction. Of the two most inveterate smokers that ever came to our knowledge, one died of starvation from inability to swallow food; the other was always smoking, always sick, when being sent to the penitentiary from Wall street, he gained fifteen pounds in three months, on the strength of entire compulsory abstinence from the "weed."

